# Avalanches in a Stochastic Model of Spiking Neurons

**DOI:** 10.1371/journal.pcbi.1000846

**Published:** 2010-07-08

**Authors:** Marc Benayoun, Jack D. Cowan, Wim van Drongelen, Edward Wallace

**Affiliations:** 1 Department of Pediatrics, University of Chicago, Chicago, Illinois, United States of America; 2 Department of Mathematics, University of Chicago, Chicago, Illinois, United States of America; 3 Computation Institute, University of Chicago, Chicago, Illinois, United States of America; University College London, United Kingdom

## Abstract

Neuronal avalanches are a form of spontaneous activity widely observed in cortical slices and other types of nervous tissue, both *in vivo* and *in vitro*. They are characterized by irregular, isolated population bursts when many neurons fire together, where the number of spikes per burst obeys a power law distribution. We simulate, using the Gillespie algorithm, a model of neuronal avalanches based on stochastic single neurons. The network consists of excitatory and inhibitory neurons, first with all-to-all connectivity and later with random sparse connectivity. Analyzing our model using the system size expansion, we show that the model obeys the standard Wilson-Cowan equations for large network sizes (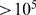 neurons). When excitation and inhibition are closely balanced, networks of thousands of neurons exhibit irregular synchronous activity, including the characteristic power law distribution of avalanche size. We show that these avalanches are due to the balanced network having weakly stable functionally feedforward dynamics, which amplifies some small fluctuations into the large population bursts. Balanced networks are thought to underlie a variety of observed network behaviours and have useful computational properties, such as responding quickly to changes in input. Thus, the appearance of avalanches in such functionally feedforward networks indicates that avalanches may be a simple consequence of a widely present network structure, when neuron dynamics are noisy. An important implication is that a network need not be “critical” for the production of avalanches, so experimentally observed power laws in burst size may be a signature of noisy functionally feedforward structure rather than of, for example, self-organized criticality.

## Author Summary

Networks of neurons display a broad variety of behavior that nonetheless can often be described in very simple statistical terms. Here we explain the basis of one particularly striking statistical rule: that in many systems, the likelihood that groups of neurons burst, or fire together, is linked to the number of neurons involved, or size of the burst, by a power law. The wide-spread presence of these so-called avalanches has been taken to mean that neuronal networks in general operate near criticality, the boundary between two different global behaviors. We model these neuronal avalanches within the context of a network of noisy excitatory and inhibitory neurons interconnected by several different connection rules. We find that neuronal avalanches arise in our model only when excitatory and inhibitory connections are balanced in such a way that small fluctuations in the difference of population activities feed forward into large fluctuations in the sum of activities, creating avalanches. In contrast with the notion that the ubiquity of neuronal avalanches implies that neuronal networks operate near criticality, our work shows that avalanches are ubiquitous because they arise naturally from a network structure, the noisy balanced network, which underlies a wide variety of models.

## Introduction

Neurons in the central nervous system are organized into recurrent networks which function dynamically, firing action potentials over time in a variety of spatiotemporal patterns. Such networks not only respond to external input, but spontaneously produce patterns of activity. Such spontaneous activity in isolated pieces of cortex has been studied since the work of B. DeLisle Burns in the early 1950s [1]. The main result was that surgically isolated parietal cortex remained silent but excitable. A sufficently strong depolarization of a site on the surface elicited a sustained propagating response, with an all-or-none character, characteristic of an excitable medium. Such a medium has a threshold for excitation.

Recently, the behavior of isolated cortical slices near or at threshold was studied systematically by Beggs and Plenz [2]. They used rat somatosensory cortex, either in mature organotypic cultures, or else in acute slices, using an 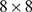 microelectrode array to record local field potentials. The slices were silent until stimulated with the excitatory neurotransmitter NMDA, in combination with a dopamine 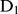-receptor agonist, whereupon they produced bursts of activity in the form of local field potentials recorded at microelectrodes.

The main result of their experiments is that these bursts of activity are *avalanches*, which [2] defines as follows. The configuration of active electrodes on the array during one time bin of width 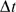 is called a *frame*, and a sequence of frames preceded and followed by blank frames is called an avalanche. The *size* of an avalanche is the total number of electrodes activated between the blank frames. The weak correlations between successive frames show that avalanche activity is neither wave-like nor periodic. Electrode activations, while appearing to be temporally coincident on a long time scale, are roughly self-similar, as can be seen by their distinct activation times when observed at smaller time scales. This is a form of synchrony, in that electrodes are more likely to be activated closer in time to activity in other electrodes. The avalanche size distribution is close to a power law, meaning that for a wide range of sizes, the probability that a given avalanche has size 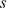 is proportional to 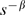.

Neuronal avalanches, by which we mean irregular synchronous activity with a power law burst-size distribution, have since been studied extensively. Avalanches have been observed not only in rat cortex *in vitro* but also *in vivo* [3], in organotypic cultures [4], [5], leech ganglia [4], and in the cortex of awake macaque [6], and used to draw inferences regarding information transmission [7].

### A theory of avalanche formation

In what follows we provide a theory for the formation of avalances using a stochastic version of the sigmoid rate model originally introduced to represent individual neural activity [8]. We call this the stochastic rate model [9]–[11]. Each neuron spikes with a probability per unit time dependent on its total synaptic input, while the resulting spiking activity decays at a constant rate. The stochastic nature of the model allows for efficient simulation via the Gillespie algorithm [12], an event-driven method.

We extend the stochastic rate model to explicitly deal with coupled excitatory and inhibitory populations. We show that this model, with appropriate connectivity, produces avalanches in an all-to-all connected network of excitatory and inhibitory neurons when a parameter is increased. We call this parameter the *feedforward strength*, 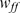 [13], since it measures the extent to which our recurrent network functions analogously to a feedforward network.

Analytically, we show that the stochastic rate model may be treated as a stochastic perturbation of the deterministic Wilson-Cowan equations [14], [15]. The stochastic rate model produces avalanches in a range of network sizes, for example thousands of neurons, depending on the parameters; in the limit of large network size, the model obeys the Wilson-Cowan equations exactly, which do not themselves produce avalanches. This analysis allows us to address the relation of avalanche dynamics to other parameters, in particular the network size and the external input to the network, showing that these dynamics are robust to wide-ranging variations in these parameters. Finally we obtain avalanche dynamics in a network with random sparse connectivity by generalizing the notion of feedforward strength.

## Results

### Individual neurons as input-dependent stochastic switches

**Figure 1:**
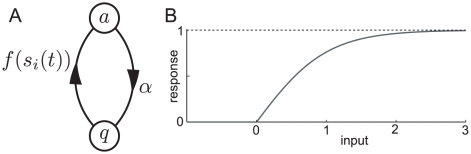
Single neuron dynamics. A, single-neuron state transitions, with the transition rates marked; for the 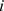
th neuron, the total synaptic input is the sum of network input and external input, 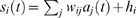. B, graph of the response function 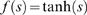 for 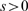.

The stochastic rate model treats neurons as coupled, continuous-time, two-state Markov processes (figure 1A); this may be seen as analogous to a deterministic neuron with very noisy synaptic input, but is agnostic about the source of the noise. Each neuron can exist in either the *active* state 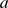, representing a neuron firing an action potential and its accompanying refractory period, or a *quiescent* state 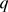, representing a neuron at rest. In order to fully describe this two-state Markov process, it is only necessary to specify the transition rates between the two states. The transition probability for the 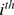 neuron to decay from active to quiescent (right arrow of figure 1A) is
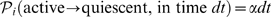
(1)as 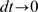, where 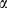 represents the decay rate of the active state of the neuron. The transition probability for the 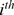 neuron to spike (left arrow in figure 1A), i.e. change from quiescent to active, is
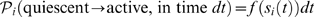
(2)

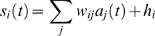
(3)as 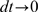. Here 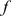 is the *response function*, giving the firing rate as a function of input, and 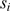 the total synaptic input to neuron 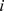, a sum of external input 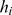 and network input 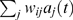, where 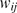 are the weights of the synapses, and the activity variable 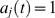 if the 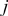
th neuron is active at time 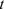 and zero otherwise.

Although there is no explicit refractory state in the model, in all simulations, 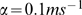, corresponding to an active state with a time constant of 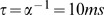 (1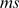 for the action potential plus 9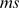 to approximate a refractory period where neurons are hyperpolarized). This choice of 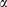 constrains neuronal firing rates to be no greater than 100 Hz.

All neurons are chosen to have the same response function,
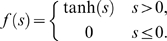
(4)As shown in figure 1B, this standard choice of response function models a neuron's firing rate as zero if it is below threshold, growing close to linearly with the synaptic input as it passes threshold, and then saturating at a maximum rate further above threshold. Since we are studying spontaneous activity in this study, external input is positive but small, so that even in the absence of any network activity, some neurons have a non-zero firing rate.

### Population dynamics evolve according to the population master equation

**Figure 2:**
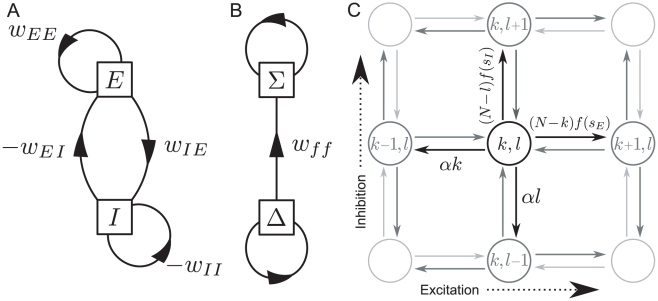
Network connectivity and dynamics. A, schematic of connection strengths between excitatory, 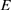, and inhibitory, 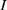, populations, where an arrow indicates a synaptic input. B, schematic of functionally feedforward connectivity, where one mode of network excitation, 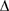, excites another mode 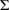, but 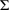 does not directly affect 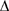. C, network dynamics visualized. If there are 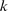 excitatory and 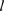 inhibitory neurons active, another excitatory neuron may become active, and network state moves rightwards one spot, at net rate 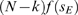, where 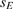 is the total synaptic input to an excitatory neuron. The rates for other transitions are shown with solid arrows and discussed in the population dynamics section of the results. Dashed arrows represent transitions into the state 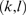 from adjacent states.

We next consider networks of excitatory and inhibitory neurons, initially with all-to-all connectivity depending only on the cell type; at the end of the results section we address how our findings extend to sparse or inhomogenous connectivities. The outgoing synaptic weight from each excitatory neuron to each excitatory neuron is 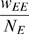, from excitatory to inhibitory is 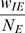, from inhibitory to excitatory is 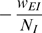, and from inhibitory to inhibitory is 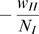. The effect is of one excitatory and one inhibitory population, connected with strengths shown in figure 2A.

The network's stochastic evolution can be thought of as a random walk between states with 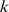 excitatory and 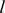 inhibitory neurons active, where the number of active neurons can increase or decrease only by one at a time, causing the state to wander around on a lattice as shown in figure 2C. Solid lines show movements out of the state 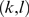 and dashed lines movements into 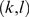. The rightwards (upwards) arrow is the result of a single excitatory (inhibitory) neuron firing in response to its synaptic input. The leftwards (downwards) arrow is associated with the decay of an excitatory (inhibitory) neuron from active to quiescent, reflecting the single neuron dynamics shown in figure 1A.

To treat this analytically, we consider the probability 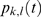 that there are 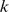 excitatory, and 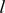 inhibitory neurons active at time 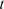. The random walk on the lattice depicted in in figure 2C is reflected by 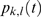 evolving dynamically in time for each state 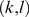. The probability 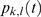 evolves according to the master equation (19). The equation and its derivation are detailed in methods; in fact the equation contains exactly the same information as figure 2C. This is a generalization of the one population master equation for the stochastic rate model introduced in [9]. Note here that, in the case of identical single neurons and all-to-all connectivity the population-level master equation is an exact description of the network evolution; if the single neuron parameters and the connection strengths were drawn from probability distributions, we would have to average over these distributions to get an approximate population-level master equation.

We use the Gillespie algorithm [12], an event-driven method of exact simulation, for all simulations of the master equation (see methods).

### How avalanches are obtained

**Figure 3:**
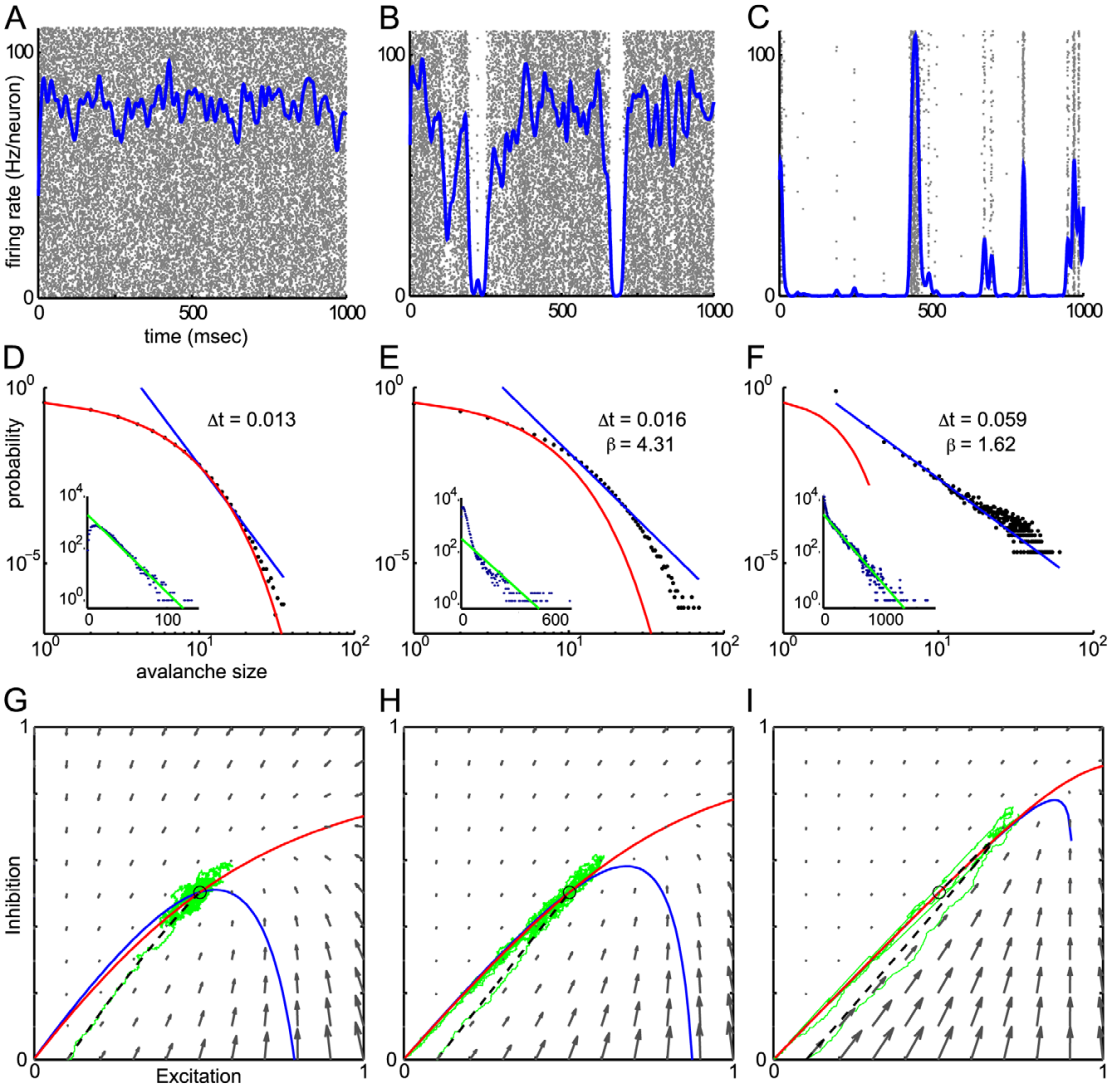
Transition from asynchronous firing to avalanche dynamics. Simulations with parameter values 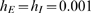, 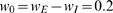, and 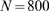. Left column, 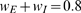, middle column, 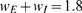, right column 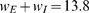. A,B,C: Mean firing rate of network (see Procedures) plotted over raster plot of spikes. Individual neurons correspond to rows, and are unsorted except that the lower rows represent excitatory neurons and the upper rows inhibitory. D,E,F: Network burst distribution in number of spikes, together with geometric (red) and power law (blue) fit; 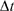, the mean inter spike interval, is the time bin used to calculate the distribution, and 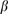 is the exponent of the power law fit. Inset, inter-spike interval (ISI) distribution in 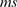 for a sample of 50 neurons from the network, shown in semi-logarithmic co-ordinates, with exponential fit (green). G,H,I: Phase plane plots of excitatory and inhibitory activity showing the vector field (grey) and nullclines 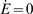 (red) and 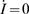 (blue), of the associated Wilson-Cowan equations and plots of a deterministic (black dashed) and a stochastic (green) trajectory starting with identical initial conditions. Note that the deterministic fixed point (black circle), where the nullclines cross, does not change as 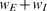 increases, but the angle between the nullclines becomes increasingly shallow, and the stochastic trajectory becomes increasingly spread out. See also figure S1.

We now investigate the range of parameters for which the stochastic model exhibits a transition from independent firing to irregular bursts of synchronous activity, i.e. to avalanches. We vary both inhibitory synaptic strength 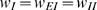 and the excitatory strength 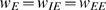, while fixing the difference between them, 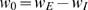. We keep the other parameters constant; as we will later show, this has the effect of leaving the deterministic equilibrium or fixed point unchanged. As shown in figure 3A, when the total synaptic strength is small, firing rates fluctuate weakly about the fixed point predicted by the deterministic Wilson-Cowan equations, meaning that the neurons fire asynchronously. The neurons fire roughly as independent Poisson processes, as shown by their approximately exponential inter-spike-interval distribution in the insets to figure 3D. The distribution of burst sizes shown in figure 3D fits a geometric distribution consistent with independent Poisson firing, explained in methods.

As we increase the synaptic input, fluctuations in the firing rate grow, and we begin to see large and long-lived downwards fluctuations away from the deterministic value of the firing rate, at random times, shown in figure 3B–C. Episodes of near-zero firing interpose between episodes of collective firing of many neurons across the network. Looking at the statistics of these irregular bursts of synchronous activity, we find that the distribution of burst sizes, measured in number of spikes, approaches a power law distribution as the firing becomes more synchronized, shown in figures 3E–F. This is therefore a candidate mechanism for neuronal avalanches [2], [4], [5]. In figure 3F, we see that the size distribution conforms to a power law for avalanche sizes between roughly 5 and 500 spikes. Testing the goodness of fit using ordinary least-squares linear regression on the bilogarithmically transformed co-ordinates, the test of significance used in [2], we find the 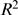 value was 0.968. However, recent research has shown that to be is an inappropriate and unreliable method for detecting power laws [16], a point we return to in the discussion. Using the maximum likelihood estimator developed in [16] (see methods) we find an exponent of 1.62. However, the goodness of fit test also developed in [16], we reject the null hypothesis that the sample is drawn from an exact power law, for its entire range, with 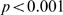.

Considering the population activity, (i.e. the proportion active per population, as opposed to the spike firing rate), figure 3G–I show that the activity also becomes increasingly prone to large fluctuations towards zero, despite the associated deterministic Wilson-Cowan equations having an unchanging single stable fixed point.

### Avalanches result from strong feedforward dynamics

We illuminate this behaviour with the help of the system size expansion [17]–[20], a standard technique from stochastic chemical kinetics, reviewed in Text S1. The inspiration for this comes from a Gaussian approximation: if the neurons were to fire independently of each other, then the total activity in each population would be Gaussian with mean proportional to 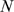 and standard deviation proportional to 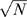. Accordingly, we model the number of neurons active at a given time 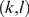 as the sum of a deterministic component 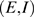, scaled by 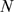, and a stochastic perturbation 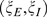, scaled by 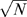, so that
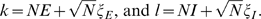
(5)The deterministic terms obey the Wilson-Cowan equations
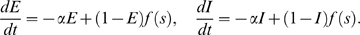
(6)where 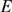 and 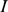 are, respectively the (time-averaged) proportions of excitatory and inhibitory neurons active in a given time bin, [see [14]], and now the total synaptic inputs are the same to both populations, 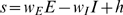, where 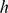 is external input. The fluctuation variables 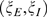 obey a linear stochastic differential equation
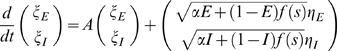
(7)to order 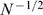, where the matrix 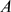 is the Jacobian of (6) calculated at the deterministic trajectory, and 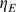 and 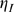 are independent white-noise variables whose amplitude is also calcuated via the deterministic trajectory. Since this equation is linear, the fluctuations are approximately Gaussian for large 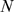. Notice that in figure 3G the trajectory of the master equation closely tracks the trajectory of the Wilson-Cowan equations (6). In the case of independent firing, the fluctuation term is small, but we see in figures 3H–I that as the network transitions to synchronous firing the fluctuations dominate and the stochastic trajectories move away from those for the deterministic system.

It is is easier to understand the dynamics by making a change of variables; to motivate this change of variables, note that large fluctuations tend to occur increasingly as inhibition approaches excitation, 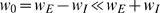. This is sometimes called a balanced network [13], [21], [22], in the sense that inhibition balances excitation. In this case, we can express the synaptic input in terms of the mean 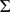 and difference 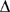 of the excitatory and inhibitory population activities, and note that the neuronal response is highly sensitive to changes in the difference and relatively insensitive to changes in the mean, described schematically in figure 2B. More precisely, if
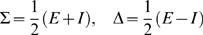
(8)then the total synaptic input is
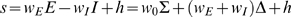
(9)where 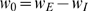. From (9) we deduce that, in the balanced case where 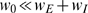, the input is much more sensitive to changes in the difference than in the mean. Accordingly, we make a linear change of variables from 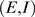 to 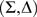. As shown in Text S1, this leads to the more transparent deterministic equations
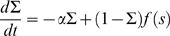
(10)

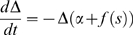
(11)with unique stable solution 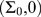. The factor of 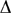 in (11) means that 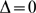 at the fixed point, and that close to the fixed point 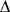 is only weakly sensitive to changes in 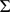. Since 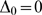, and 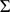 depends on the sum of the weights only through the term 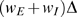 which is zero at the fixed point, in fact the fixed point 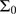 is left unchanged by varying the sum 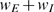 while keeping the difference 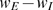 constant. This is why the fixed point is the same in figures 3G–I.

In these new variables the linear noise approximation [see Text S1] is expressed as
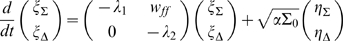
(12)where 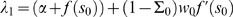, 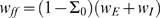 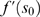, 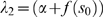, and 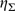 and 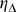 are again independent white-noise variables. The Jacobian matrix
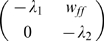
(13)is upper-triangular, and has eigenvalues 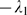 and 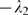. If 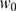 is small and positive, so are the eigenvalue magnitudes 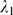 and 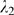. To see this, note that 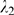 is the sum of two small terms 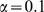 and 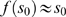; the extra term in 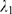 is also small if 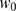 is small, since 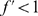. Thus, the fixed point is weakly stable, and like the location of the fixed point, its linear stability depends on the weights only via the difference 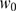.

The off-diagonal term 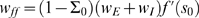 has been called a hidden feedforward term [13], [23]–[25], feedforward because fluctuations in 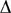 feed into the evolution of 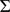 but not vice versa, and hidden because a change of variables is required to see this structure, not obviously present in the network connectivity (figure 2B). The Jacobian, with small eigenvalues but a large off-diagonal term, leads to the amplification of small values of 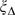 into transient increases in 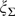 whose magnitude increases with 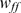. This effect is called *balanced amplification* in [13]; it may also be thought of as a shear flow in the phase plane, and is characterized by the nullclines crossing at a shallow angle. In figures 3G–I, one can see that the nullclines become closer to parallel as the feedforward term 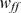 increases.

In a noisy system, the functionally feedforward mechanism means that small spontaneous fluctuations in 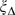 are amplified into transient increases in 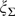 whose size increases with 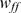. An appropriate combination of the noise being strong enough, the feedforward term 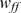 being large enough, and the eigenvalue damping the fluctuations 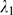 being small enough, leads to large sustained fluctuations in 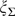.

We may make this more explicit by examining the variance of the activity, calculated in Text S1, from the linear noise approximation as
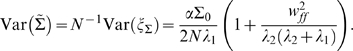
(14)Fluctuations predicted by the linear noise approximation grow with the strength of the functionally feedforward term, and also grow as the eigenvalues 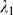 and 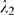 go to zero.

We may relate the above findings to the fluctuations in firing rate found in simulations, by observing how the mean and standard deviation of the time-binned spike count varies as we increase the feedforward strength 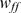. We time bin the spike counts into bins of width 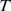, so that the number of spikes in the 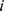
th bin is 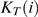. Then the normalized firing rate is 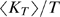 and the normalized standard deviation is 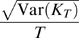.

**Figure 4:**
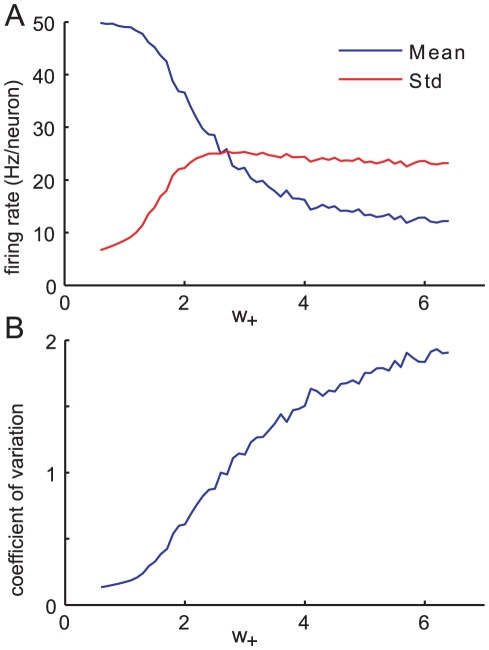
Activity and synchrony for a range of feedforward strengths. A: Mean and standard deviation of time-binned firing rate and; B: coefficient of variation plotted against the sum of synaptic weights 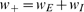, from simulations with other parameters fixed, 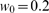, 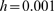 and 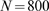. The timebin width is 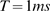. Note that the feedforward strength 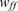 is proportional to the sum of weights, 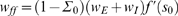.

In figure 4A we see that as the feedforward strength increases, the standard deviation initially increases sharply. Meanwhile, the mean firing rate drops, and continues to drop even as the standard deviation saturates. The effect of this is that the coefficient of variation (CV), 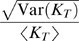, which measures the typical size of the fluctuations relative to the mean, increases, initially rapidly but later more slowly, as shown in figure 4B. (Note that this is the CV of the time-binned spike counts, not the much studied CV of the inter-spike interval.)

The linear noise approximation, via equation (14), predicts the increase in the standard deviation with 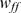. Although the linear noise approximation predicts no change in the mean, correction terms at the next order, 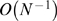, indicate that the mean decreases as 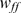 increases (see Text S1). This leads to the counterintuitive observation that the deterministic fixed point does not even accurately describe the mean value of the stochastic system when fluctuations are large.

**Figure 5:**
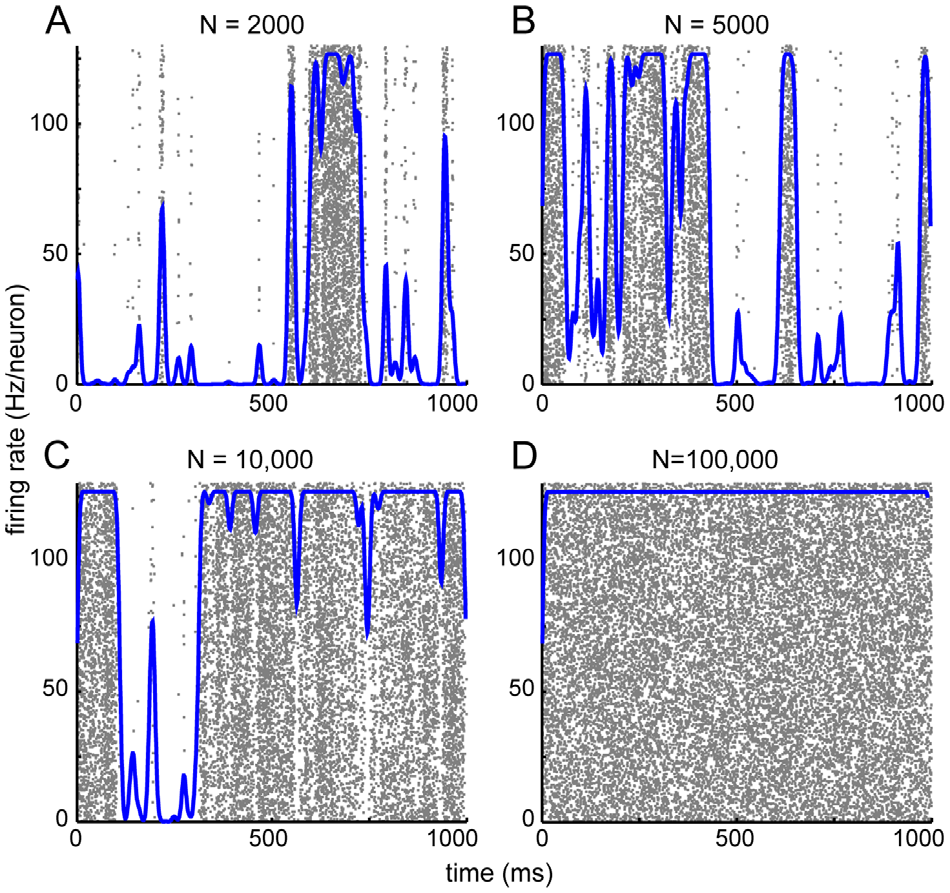
Avalanches persist for intermediate network size and are extinguished at larger sizes. Effect of varying the size per population, 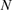, with other parameters fixed as 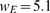, 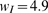, 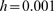. A: N = 2000. B: N = 5000. C: N = 10,000. D: N = 100,000.

Another prediction from (14) is that the fluctuations become small as 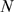 increases, in particular causing the firing rate to return to its deterministic limit. In figure 5 we show the effect of varying the size of the network. Fluctuations do indeed die away at large size, and the firing rate barely fluctuates for 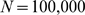 neurons per population; however, irregular bursts are still observed in networks with size of up to 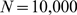 neurons per population. This indicates that, although the stochastic Wilson-Cowan model has as its large-scale limit the deterministic Wilson-Cowan equations, the network size may need to be extremely large for the deterministic equations to accurately describe its behavior.

### Response to changing input

**Figure 6:**
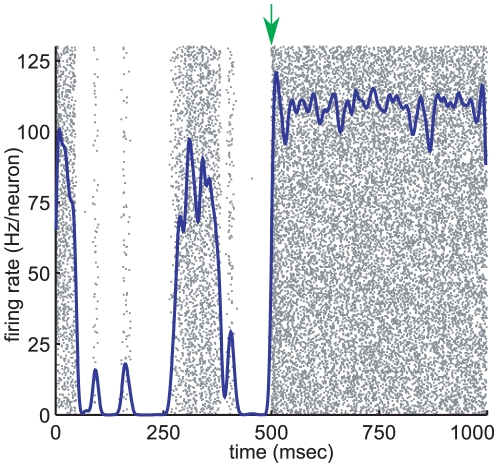
Response of network to change in input. Here, the constant input is 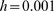 for the first 500ms and 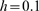 for the following 500ms; the change is indicated by the green arrow. The other parameters for this all-to-all network are, 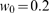, 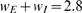, and 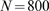.

We have found spontaneous dynamics organized into irregular synchronous bursts in neural networks with very weak constant input. To shed light on how networks of neurons process information, we want to know what happens when the input varies. In the simplest case - where input 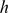 to every neuron is identical, but may change over time - a change in the magnitude of 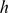 alone may be sufficient to cause the network to move from irregular to regular behaviour, shown in figure 6. Here a change in the input strength makes the fixed point more stable, so decreases the extent to which the network at the fixed point is functionally feedforward.

We can see this by tracking the changes caused in the Jacobian matrix (12) at the fixed point with respect to the mean and difference co-ordinates 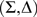. Increasing the external input 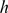 results in an increase in the synaptic input 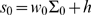, both directly as 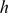 appears in the sum, and indirectly as it causes the fixed point 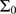 to increase. This causes the eigenvalue 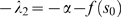 to become more negative, increasingly the stability of the fixed point. Since the response function saturates, so has a decreasing derivative, the other eigenvalue 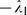 also becomes more negative as input increases. Similarly the feedforward term 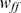 decreases. In other words, when input is high, spontaneous internal network correlations quickly decrease. This quick response to an increase in input is a computationally desirable property previously observed in balanced networks [13], [21], [22].

The effects of altering various parameters of the model starting from independent firing are summarized in table 1, where an increase in the coefficient of variation means that fluctuations are proportionately greater, or that the dynamics are more avalanche-like.

**Table 1:** The effect of changing system parameters on the mean, variance, and coefficient of variation, estimated from the linear noise approximation.

parameter	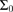	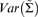	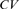
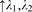	-	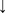	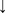
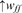	-	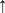	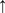
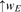	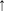	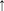	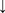
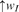	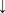	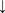	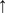
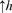	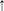	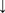	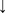
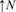	-	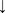	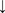

### Sparse connectivity

**Figure 7:**
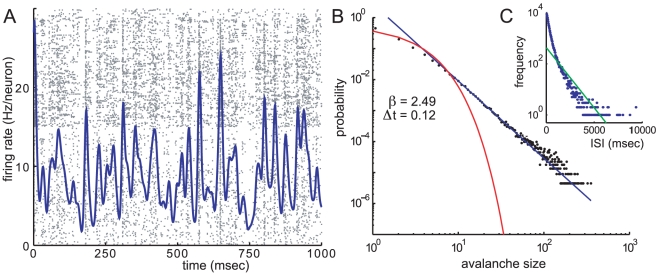
Avalanches in a sparsely connected network. Results from an excitatory and inhibitory network with 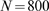, with 17% connectivity. See text for details of sparse weight matrix. A: Raster plot and mean firing rate. B: Avalanche size distribution, calculated with bin size 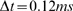 and showing poisson fit (red) and power law fit (blue) with exponent 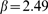. C: Inter-spike-interval distribution with exponential fit (green).

The number of synapses per neuron in cortex is believed to be at most 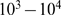 [26], so only networks with fewer than 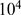 neurons could have anything approaching all-to-all connectivity; larger networks in cortex must be sparsely connected. Our results so far deal with all-to-all connected networks, so it is reasonable to ask whether or not a sparsely connected network could produce avalanches via the same mechanism. The answer is yes: we are able to generate random sparse matrices with weakly stable fixed points and high functional feedforward connectivity which exhibit large fluctuations grouped into avalanches, as shown in figure 7.

We used the same single-neuron parameters and response function as the all-to-all case, changing only the connectivity matrix. To make this matrix, we generated random sparse positive matrices 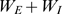 with large eigenvalues, and 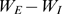 with small eigenvalues, so that the weight matrix
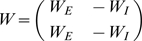
(15)is random, sparse and obey's Dale's principle that every column, representing the synaptic weights outwards from a single neuron, is either all excitatory or all inhibitory [27]. The details of how to construct such a weight matrix are given in methods. The condition that the eigenvalues of 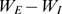 are much smaller than those of 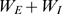 is analogous to the population condition 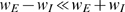 in the all-to-all case. As in the all-to-all case, this sparsely connected network has a single stable fixed point, and a change of variables to the mean and difference of the activities leads to the Jacobian at the fixed point having small negative eigenvalues and large off-diagonal elements causing strong functionally feedforward dynamics.

We conclude that homogenous all-to-all connectivity, which has the effect of averaging the population activity at the input to every neuron, is not a requirement for strongly synchronized fluctuations grouped into avalanches. The same mechanism produces similar fluctuations in an inhomogenous network if the functional feedforward strength is large enough.

## Discussion

Using the stochastic rate model in an excitatory and inhibitory network, we simulated avalanche dynamics, or irregular synchronous activity with a power law burst size distribution. We showed that a network's propensity to produce such bursts depends on it having a functionally feedforward structure described by [13], in the presence of noise. This is achieved by making the network balanced, meaning that the net difference between excitation and inhibition is small compared to the sum of excitation and inhibition. Bursts arise from small spontaneous fluctuations in the difference of excitatory and inhibitory activity, amplified by the functionally feedforward structure into large fluctuations in the activity of both populations. We demonstrated avalanche dynamics to be robust over a wide range of system parameters. Depending on the functional feedforward strength, this fluctuation-driven behaviour persists in networks of at least tens of thousands of neurons. Increasing the network size (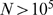 neurons, depending on other parameters) causes the network to fire asynchronously at a rate given by the deterministic Wilson-Cowan equations. However, the deterministic equations do not produce avalanches and exhibit no qualitative change in their spontaneous dynamics due to functionally feedforward connectivity, unlike the stochastic rate model. A significant increase in the external input quickly moves the stochastic network out of the functionally feedforward regime, also causing the network dynamics to behave more like the deterministic Wilson-Cowan equations. Avalanche dynamics are also robust to major changes in the synaptic connectivity - we can produce such fluctuations in a sparse randomly connected network by constraining the eigenvalue spectrum of its connectivity matrix so that it has a functionally feedforward structure.

### Limitations of our findings

Although simplified models are commonly used to study neural network dynamics, the question remains whether a given simplification is appropriate for modeling the network at hand. Our model neurons, which are stochastic switches, are so simple as to make it difficult to relate their parameters precisely to the cells being modeled, although not as difficult as for a purely population-based model. Two-state Markov processes have been previously used for modeling neurons at longer timescales, for example the states representing a zero or nonzero firing rate in studies of attractor networks [28], or up and down states in cortex in studies of repeating patterns of activity [29], contrasting with our use of a state transition to represent a single spike. Such simple stochastic models may produce qualitatively the same network dynamics as more biophysically detailed models, while their simplicity enables them to give insight into the mechanisms of emergent phenomena [14], [30]; we expect that further research will show the same to hold for our model. In addition, it would be interesting to see if functionally feedforward connectivity could produce avalanche dynamics at much longer timescales via the model of Roxin et al. [29].

Another concern is that the time scales in our simulations reflect the time scales in cortex. For example our cellular firing rates are at the high end of those observed in cortex in the asynchronous case. One simple way to adjust our model is to place a time constant 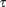 in front of the time derivative term in the master equation, or equivalently to scale all the transition rates by 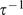, thus slowing down the entire simulation, including firing rates, by a constant factor. One could also scale the transition rates differently for each population, since excitatory neurons tend to have lower firing rates than inhibitory neurons in cortex [31]. Another way to slow down the rate of occurence of avalanches without changing the single-neuron parameters is, by increasing the size of the simulated network to match the size of a cortical slice, so decreasing the effective noise strength which is proportional to the square root of the size. Since the avalanches are noise-driven fluctuations, with appropriate adjustments to the connectivity parameters this would make the time between avalanches longer.

The lack of conduction delays in our model raises another issue with the time scales: the delay in activation of one neuron by another is accounted for solely by the random exponential time to spiking, thus meaning that a postsynaptic spike may follow a presynaptic spike at a delay shorter than is reasonable for causality in cortex. We would expect the introduction of delays to slow down the network dynamics, and also be relatively straightforward to simulate as an adaptation of the Gillespie algorithm to account for delays already exists [32]. As neurons in larger networks are more likely to be far apart, we might expect conduction delays to play a bigger role in larger, spatially distributed networks.

Although we showed that self-organization is not needed to maintain avalanching dynamics in a network, this begs the question, what kind of self-organization can put the network in a regime where it produces avalanches? In cortical cultures from layers 2/3 of the rat, avalanche-like dynamics emerge after 6–8 days [3]; similarly, in cultured networks of dissociated rat hippocampal neurons, avalanche dynamics emerge after 3–4 weeks [5]. Feedforward connectivity requires the sum of excitatory and inhibitory synaptic inputs to be on average much greater than the difference, and we would expect it to take time to develop extensive enough connectivity for the total to be large. An extension of our model to involve slow modification of network properties, for example by synaptic plasticity, would be needed to account fully for these experimental results.

### Implications for experiments

If the proposed mechanism of functionally feedforward connectivity generates neuronal avalanches in an experimental system, it should be possible to probe that system in ways analogous to varying the parameters in our model. For example, the model predicts no activity in the absence of external input, since the only fixed point of the model is the origin. If the network topology already exhibits strong feedforward strength, then the addition of small concentrations of an excitant would effectively increase the external input parameter, so shifting the fixed point away from the origin and causing avalanches. This was in fact the method used by Beggs & Plenz [2], who added NMDA (N-methyl-D-aspartic acid) to produce avalanches in cortical slices and cultures. If too much NMDA is added, however, then we expect an excess of excitation, so that the near balance of excitation and inhibition responsible for the strong feedforward strength of the network would be disrupted and avalanches would no longer occur.

A small increase in extracellular 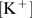 effectively increases both excitatory and inhibitory synaptic weights, thereby increasing the feedforwardness 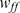 while keeping the difference 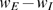 relatively unchanged, leading to increased burst frequency in our model. This suggests that an experimental preparation could be studied near the avalanche transition by titrating with 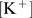. If the network were in a state where 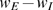 is slightly positive, as in the simulations performed here, then further addition of a small amount of an inhibitory antagonist such as bicuculline (a 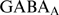 antagonist) would weaken 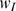, thereby increasing the difference 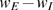 and leading counterintuitively to decreased burst frequency after the addition of an inhibitory blocker. If the synaptic weights were initially elevated by increasing extracellular 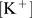, this would ensure the feedforwardness to be much larger than the difference 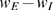, so that weakening 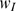 would make a proportionately larger change to the difference. This may be the effect at work in [33], where adding 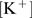 and bicuculline together produced a lower overall burst frequency than adding potassium alone, in a slice preparation of rat hippocampus.

If it were possible to add carefully co-ordinated amounts of an inhibitory blocker and an excitatory blocker, the model raises the possibility that a network, by becoming less functionally feedforward, could have higher mean firing rates but fewer bursts. In general, if there are pharmacological manipulations corresponding to varying the parameters as shown in table 1, we expect the coefficient of variation of the firing rate, our proxy for the strength of avalanche dynamics, to move accordingly.

### Relation to previous modeling work

#### The role of noise

In a well-known paper, van Vreeswijk and Sompolinsky [22] described the activity of a network of excitatory and inhibitory integrate-and-fire neurons with sparse random connectivity. They ensure that spontaneous network activity is driven by internal fluctuations by arranging that excitation and inhibition are balanced, meaning that both the mean and the standard deviation of net synaptic input scale as 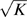, where 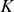 is the mean number of connections per neuron, and also scaling the threshold of each neuron with 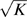.

By contrast, the present study keeps the thresholds fixed while scaling the connection strengths inversely with network size. This means that the mean synaptic input scales with the threshold, as a constant; the fluctuations scale as 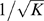 in the case of independent firing, but become comparable to the mean input when the network synchronizes. The analysis of [22] relies on firing of neurons being weakly correlated, achieved via static randomness in the weight matrix. The stochastic rate model instead undergoes a transition between uncorrelated and correlated firing achieved via random spike times of individual neurons. Other key dynamical features of the [22] model, that without inhibitory input a neuron fires at a high rate, but without excitatory input neurons are very unlikely to fire, are also found in our model. Thus we achieve similar ends with very different modeling assumptions, in our case relying on the network's ability to self-synchronize and on a different source of disorder.

#### The interpretation of power laws

The present paper interprets a power law distribution to mean that for a wide range of sizes, the probability that a given event has size 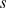 is proportional to 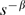. The avalanche size distribution in figure 3F indicates a good power law fit for roughly two orders of magnitude. Since biological systems are finite and measurements have limited resolution, we don't expect observed power laws to extend all the way to infinity. Power law behaviour with a small exponent, holding over significantly more than one order of magnitude, is enough to show that a phenomenon crosses several spatial or temporal scales. This is a sufficient condition to label a dataset as a power law in this loose sense, a widely shared interpretation [34].

The analysis of such data is complicated by the lack of appropriate statistical tools to estimate and test them. Since a power law distribution corresponds to a straight line in bilogarithmically transformed co-ordinates, it would be tempting to use ordinary least squares linear regression analysis to calculate the slope of this line and assess the fit through the coefficient of determination. However, this analysis is ill-founded and the estimate of the slope of the power law is biased, as discussed thoroughly in [16]. This method of comparison was used in experimental studies of avalanches [2], and so we analyzed our simulated data with the same method for comparison. Clauset et al [16] also developed a maximum likelihood estimator and test for data distributed according to an exact power law; in this paper we use their estimator. More recent studies have found that cortical avalanche data from awake cat do not follow an exact power law according to these newer tests [35]. We are not aware of a well-developed goodness of fit test for data conforming to a power law distribution for a finite range of values; the development of such tests would be very helpful for further research in the area.

#### The mechanism of avalanche formation

A variety of models have attributed avalanches to criticality in network dynamics [36]–[38], meaning that avalanches occur when the network lies on the boundary between stability and instability. In the language of dynamical systems, this would mean that avalanches occur when an eigenvalue equals zero. This situation is extended by our model, which exhibits avalanches not only when the eigenvalues are close to zero, but when the eigenvalues are small relative to the feedforward strength. Consequently, it is not possible to infer criticality in a network from the fact that it exhibits large fluctuations whose size obeys a power law distribution.

Some models for neuronal avalanches have further suggested an underlying mechanism of self-organized criticality [38]. This would mean that the network has some fast-changing dynamical variables, for example firing rate, which are maintained at the boundary of stability and instability by the movement of slow variables, such as synaptic plasticity or dendritic growth [39]. Because such systems often have power law statistics, the measurement of power laws is sometimes taken as evidence for an underlying self-organized critical mechanism [40]. Our model has neither critical behavior in the fast variables, nor slow variables to modulate their dynamics, yet has the characteristic power law distribution of fluctuation size. Criticality is a sufficient but not necessary condition for the emergence of power laws.

Since the slope of the power law observed is dependent on the choice of bin width in experimental results [2], and also in our model (figure S1), we do not read any significance into the particular slope observed. This makes us skeptical that an observed slope of 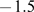 in a neural network, imported from directed percolation or critical branching models [7], [10], [37], actually results from one of those models underlying the phenomenon. In our view, any good model of neuronal avalanches must reproduce the variability in the observed slope of the power law with temporal bin width.

Our connectivity is homogenous, or sparse and random. Another model used scale-free network connectivity, where the number of synaptic connections per neuron has a power law distribution, to generate power law distributed activity [4]. It is not surprising that such a long-tailed distribution in the connectivity results in a long-tailed distribution in the firing; however, our model produces a power law distribution of activity without requiring a power law distribution in the connectivity.

Since functionally feedforward dynamics for a network depends only on how spectral properties of the connectivity matrix interact with the network's input via its response function, we expect these bulk dynamics to be obtainable in a variety of different connection topologies beyond the all-to-all and random connectivities examined here. Connectivity may be made more or less sparse, more or less homogenous, more or less random, may have small-world or only local topology, while the eigenvalue spectrum is constrained so that the underlying dynamics is functionally feedforward. This may explain why neuronal avalanches are observed in vastly different anatomical structures - only a few bulk properties of the network need hold, and the network will exhibit irregular synchronous firing with a long-tailed burst size distribution. Noisy functionally feedforward structure, needing neither precise tuning of the network to criticality, nor a postulated mechanism of self-organization, nor strong assumptions on the underlying connectivity, are then a simple and general mechanism for producing neuronal avalanches.

Avalanches were not observed in the functionally feedforward model examined in [13]. Their investigation was restricted to a deterministic linear firing-rate model, analogous to the linear noise approximation used here, with the same change of variables revealing the feedforward structure. Because their model exhibits a strong transient response to input, in the presence of noise it also exhibits a strong transient response to fluctuations. The linear model also required excitatory synaptic strength to be less than the inhibitory strength to maintain the stability of the fixed point, as feedback inhibition is the only ingredient there that stabilizes the strong recurrent excitation. Consequently, the linear model breaks down as the excitatory strength moves closer to the inhibitory strength, as there is no nonlinear saturation term to damp oscillations. In this sense, the functionally feedforward model without noise or saturation is not robust, as addition of small amounts of noise can drastically change its behaviour. However, with the inclusion of these terms, such a model could be used to study both spontaneous and input-driven activity.

### Conclusions

This study of neural network dynamics shows that the stochastic rate model may be viewed as a stochastic generalization of the Wilson-Cowan equations. In this context neither specific types of neural connectivity, nor tuning or self-organization to criticality, are necessary for the emergence of avalanche dynamics, namely spontaneous network bursts with power-law distributed burst sizes. What is important is that the net difference between excitation and inhibition should be small compared to the sum of excitation and inhibition, so that the network effectively has feedforward structure. Small random fluctuations, here provided by stochastic single neurons, are amplified by the functional feedforward structure into bursts involving many neurons across the network. Analogous deterministic models with functionally feedforward structure do not produce avalanches. Thus stochastic functionally feedforward networks are a sufficient and general condition for the emergence of avalanche dynamics, and a mechanism for the spontaneous production of network bursts.

## Methods

### Deriving the master equation

Here we show how to derive the master equation governing the evolution of the network state, visualized in figure 2C.

We consider 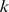 active excitatory neurons, each becoming inactive at rate 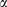. This causes a flow of rate 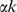 out of the state 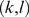 proportional to 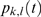, hence a term 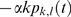. Similarly the flow into 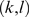 from 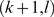, caused by one of 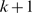 active excitatory neurons becoming inactive at rate 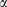, gives a term 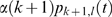. The net effect is a contribution
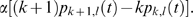
(16)In state 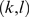, there are 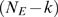 quiescent excitatory neurons, each prepared to spike at rate 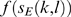, leading to a term 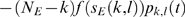, where the total input is
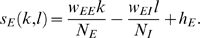
(17)Correspondingly, the flow into the state 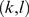 from 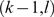 due to excitatory spikes is given by 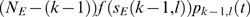. The total contribution from excitatory spikes is then
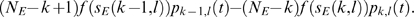
(18)There are analogous terms for the decay of active inhibitory neurons and the spiking of quiescent inhibitory neurons. Putting this together, the probability evolves according to the master equation
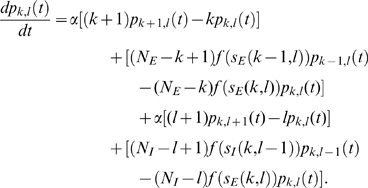
(19)


### Simulation method

We simulate the entire network as a single continuous-time Markov process, using Gillespie's exact stochastic simulation algorithm [12]. The most general form of this starts with the single-neuron transition rates, that for the 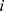
th neuron being:
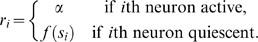
(20)The algorithm takes the state of the network, i.e. each neuron is specified as being either active or quiescent, and proceeds as:

1. Find neuronal transition rates 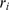, and network transition rate 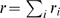.
2. Pick time increment 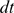 from an exponential distribution of rate 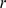.
3. Pick 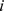
th neuron with probability 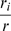, change its state, and update time to 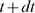.

In the case of homogenous all-to-all networks, if one only wants to simulate the number of neurons active in each population, one may simplify this algorithm along the lines of Gillespie's original presentation for a well-mixed chemical system, since the upwards transition rates 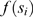 would be identical for all neurons in a population. The simplified algorithm uses much less memory and runs considerably faster.

The Gillespie algorithm is event-driven [41] in the sense that the simulation time is moved on only when the network state is updated, and the time intervals 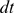 are random variables dependent upon the network state. It is then necessary to store only a vector of transition times and a corresponding vector of which neuron transitioned at each time. In the case of fluctuating firing rates found in avalanche dynamics, the algorithm, by its definition, adapts its time-steps to the firing rates, which can be a computational advantage.

All simulations were performed in Matlab 7.1 (Mathworks, Natick, MA).

### Temporal coarse-graining

To produce plots of the mean firing rate, we counted the number of spikes 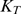 in timebins of width 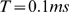, and smoothed the signals by convolving with a Gaussian of width 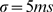. The phase-plane figures (3G–I) show an approximation to the proportion active: since active neurons decay at rate 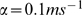, we may calculate the activity from the spike times as 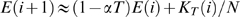.

The mean firing rate, plotted in figure 4 and over the raster plots (figures 3A–C etc.), and the activity, plotted in the phase plane figures (3G–I) and used in the calculations, are closely related. Due to the single-neuron dynamics described in (2), the firing rate, which is the rate of transitions from active to quiescent per neuron per second, is 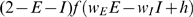 in the all-to-all case.

### Defining neuronal avalanches

We define a neuronal avalanche as a sequence of spikes such that no two consecutive spikes in the avalanche are separated by a time greater than 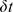. The size of an avalanche is defined as the total number of spikes belonging to the sequence. Clearly, if 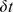 is small, then avalanche sizes will be small. Indeed, in the limiting case that 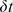 is smaller than the minimum time interval between any two consecutive spikes in the network, each spike becomes its own avalanche, so all avalanches have size unity. Similarly, if 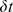 is chosen to be large, then avalanches will be large. Again consider a limiting case, where 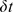 is on the order of the entire simulation time. Then all of the spikes belong to a single avalanche. We estimate an appropriate 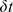, 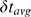 as the average time interval between consecutive spikes in the network [2], [42]. More precisely, let 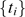 be the ordered sequence of spike times in the network, then
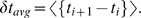
(21)This is the same as the total number of spikes in the simulation divided by total simulation time.

### Avalanche size distributions

We fit two distributions to the avalanche size. Firstly, if each neuron spikes independently as a Poisson process, then the entire network fires as a Poisson process, with a rate 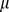. Then, the distribution of avalanche size 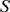 is
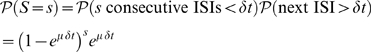
(22)which is a geometric distribution with parameter 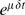. This is the red line in figure 3D–F.

It has been hypothesized that avalanche size distributions are consistent with a power law distribution, with the size 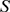 given by
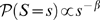
(23)for some reasonably large range of 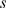. Note that this means the distribution is linear in bilogarithmic coordinates. The best fitting power law distribution to the avalanche size data was obtained by using a maximum likelihood estimator (MLE) for the slope of a power law probability distribution for discrete data (avalanche sizes are integer values only); the derivation and uses of this of this estimator are clearly explained by Clauset et al [16]. According to the MLE the slope 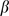 is given by the equation
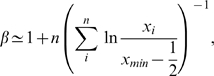
(24)where 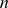 is the number of avalanches greater than size 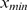 and 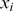 is the size of the 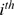 avalanche. We take 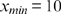 = 10. This is the blue line in figure 3D–F. Note that this is a different method for obtaining slope values than the more common ordinary least squares linear regression analysis (LRA) of the bilogarithmically transformed data. LRA is based on the assumption that the noise in the dependent variable is independent for each value of the independent variable and normally distributed. Although this is true when the dependent variable is the probability of a certain size avalanche, it does not hold after the bilogarithmic transformation. The transformed probability distribution has log-normally distributed noise, and so a calculation of the slope from LRA methods can give spurious results [16], and a biased estimate of the avalanche slope.

### Making the sparse connectivity matrix

Here we show how to make the sparse matrix with functionally feedforward connectivity; the construction is closely related to the supplementary information from [13].

For a network with 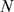 excitatory and 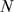 inhibitory neurons, we make a connectivity matrix
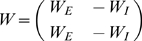
(25)The 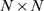 matrices 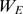 and 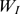 are created from random orthogonal matrices 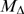 and 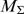 and sparse diagonal matrices 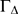 and 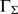, by
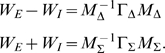
(26)Since 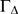 and 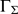 have sparse diagonal entries, we ensure that 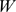 is sparse. By choosing the non-zero diagonal components of 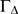 to be much smaller than those of 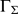, we pick the eigenvalues of 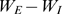 to be much smaller than those of 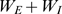; this condition is analogous to the population condition 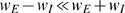 in the all-to-all case, and means that there will be a large feedforward component to the network dynamics. The fact that 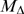 and 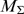 are orthonormal means that both 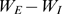 and 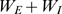 are normal, i.e. their eigenvectors are mutually orthogonal.

Next we recover 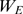 and 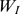 from their sum and difference in the obvious way, but adjust any negative elements of these to zero so that the resulting matrix (25) obeys Dale's principle. This perturbation of 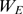 and 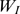 leads to a perturbation of 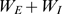 and 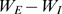, making them no longer exactly normal. A normal matrix 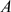 satisfies 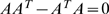, and we may measure the deviation from normalcy of 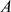 by taking the Frobenius norm, i.e. the sum of the squares of the elements, of 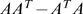. For the particular matrices under study this deviation from normalcy is very small, remaining less than 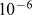 for both matrices after the perturbation.

Now we introduce a generalized Wilson-Cowan equation for the vector of neural activities 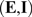 so that
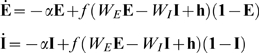
(27)where we interpret the response function as the diagonal operator 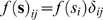.

This set of equations has a single fixed point 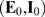 for the given weight matrix, due to the symmetry in input currents, 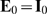. Accordingly, we change variables to sum and difference modes
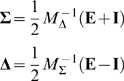
(28)so that equations (27) become
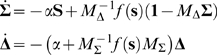
(29)where the synaptic input is 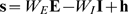 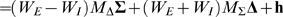.

If we replace 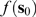 with the population average 
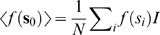
, the Jacobian of the system at the fixed point 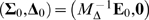 is approximated by
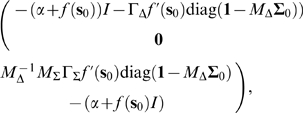
(30)which is upper triangular since 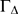 is diagonal. It can be shown that since the elements of 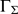 are large, the off-diagonal elements of this matrix will be much larger than the diagonal ones, leading to strong feedforward dynamics. It should be noted that if the net input current to each neuron is the same at the fixed point, as it is in the all-to-all case, then 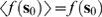, and (30) becomes exact.

## Supporting Information

Figure S1Avalanche size and duration distributions for different time bin sizes. Avalanche distributions from a single simulation with parameter values 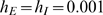, 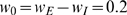, 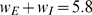, and 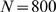. Left column, 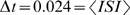; right column, 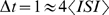. Upper graphs show the distribution of avalanche size in numbers of spikes, and lower graphs show the distribution of avalanche duration, i.e. the elapsed time between the first and last spike in an avalanche, in msec. Note that the data shows power law fit in all cases, but the slope of the distribution changes with the time bin size.(0.71 MB EPS)

Text S1 Calculations supporting main paper.
(0.10 MB PDF)
