# Supplementary material for: Avalanches in a Stochastic Model of Spiking Neurons
Source: PLoS Comput Biol. 2010 Jul 8;6(7):e1000846. doi: 10.1371/journal.pcbi.1000846 (PMC2900286; doi:10.1371/journal.pcbi.1000846)
Supplement: Text S1 — Calculations supporting main paper. (0.10 MB PDF) [file pcbi.1000846.s002.pdf]

Avalanches in a stochastic model of spiking neurons Supporting Information

Marc Benayoun[1,], Jack D. Cowan[2], Wim van Drongelen[1,3], Edward Wallace[2,*]
1 Department of Pediatrics, University of Chicago, 900 E. 57th Street, Chicago, IL, USA
2 Dept. of Mathematics, University of Chicago, 5734 S. University Ave., Chicago, IL, USA
3 Computation Institute, University of Chicago, 5735 South Ellis Avenue, Chicago, IL, USA
∗ Corresponding author: ewallace@math.uchicago.edu

S1 Calculations supporting main paper

S1.1 The System Size Expansion

Here we show how to derive the linear noise approximation in section 2.4 of the main text.

We start with the Master equation, which describes the evolution of the probability distribution for the discrete system. Let $p_{k,l}(t) = \mathcal{P}(k$ excitatory
and l inhibitory neurons are active at time $t)$. We make the simplification that $N_E = N_I = N$. Starting from the decomposition $k = N\tilde{E} = NE + N^{1/2}\xi_E$, $l = N\tilde{I} = NI + N^{1/2}\xi_I$, we derive from this equations for the evolution of the deterministic part (E, I) and the fluctuating part (ξ_E, ξ_I). The evolution equations take the form of a series with a small parameter $N^{-1/2}$.

The Master equation is then

$$\begin{aligned}\frac{dp_{k,l}(t)}{dt} = \alpha\left[(k+1)p_{k+1,l}(t) - kp_{k,l}(t)\right] \\ + \left[(N-k+1)f\left(s_E(k-1,l)\right)p_{k-1,l}(t)\right. \\ \left. - (N-k)f\left(s_E(k,l)\right)p_{k,l}(t)\right] \\ + \alpha\left[(l+1)p_{k,l+1}(t) - lp_{k,l}(t)\right] \\ + \left[(N-l+1)f\left(s_I(k,l-1)\right)p_{k,l-1}(t)\right. \\ \left. - (N-l)f\left(s_E(k,l)\right)p_{k,l}(t)\right] \quad (1)\end{aligned}$$

where the synaptic inputs are $s_E = w_{EE}E - w_{EI}I + h$ and $s_I = w_{IE}E - w_{EI}I + h$, and f is the response function. Following [1] introduce the shift operators e^{∂_k} and e^{∂_l}, which formally express

$$f(k+1) = e^{\partial_k}f(k) = f(k) + \partial_k f(k) + \frac{1}{2}{\partial_k}^2 f(k) + \frac{1}{3!}{\partial_k}^3 f(k) \ldots \quad (2)$$

So that the Master equation can be rewritten as

$$
\begin{aligned}
\frac{dp_{k,l}(t)}{dt} &= \alpha\left(e^{\partial_k}-1\right)kp_{k,l}(t) \\
&+\left(e^{-\partial_k}-1\right)(N-k)f\left(s_E(k,l)\right)p_{k,l}(t) \\
&\quad+\alpha\left(e^{\partial_l}-1\right)lp_{k,l}(t) \\
&+\left(e^{-\partial_l}-1\right)(N-l)f\left(s_E(k,l)\right)p_{k,l}(t) \\
&= -\partial_k\left[NA_E\left(\frac{k}{N},\frac{l}{N}p_{k,l}(t)\right)\right] \\
&+\frac{1}{2}\partial_k{}^2\left[ND_E\left(\frac{k}{N},\frac{l}{N}\right)p_{k,l}(t)\right] \\
&-\frac{1}{3!}\partial_k{}^3\left[NA_E\left(\frac{k}{N},\frac{l}{N}\right)p_{k,l}(t)\right]+\dots \\
&\quad-\partial_l\left[NA_I\left(\frac{k}{N},\frac{l}{N}\right)p_{k,l}(t)\right] \\
&+\frac{1}{2}\partial_l{}^2\left[ND_I\left(\frac{k}{N},\frac{l}{N}\right)p_{k,l}(t)\right] \\
&-\frac{1}{3!}\partial_k{}^3\left[NA_I\left(\frac{k}{N},\frac{l}{N}\right)p_{k,l}(t)\right]+\dots
\end{aligned}
\tag{3}
$$

where we define drift and diffusion functions

$$
\begin{aligned}
A_E(x,y) &= -\alpha x+(1-x)f\left(w_{EE}x-w_{EI}y+h\right) \\
D_E(x,y) &= \alpha x+(1-x)f\left(w_{EE}x-w_{EI}y+h\right) \\
A_I(x,y) &= -\alpha y+(1-y)f\left(w_{IE}x-w_{II}y+h\right) \\
D_I(x,y) &= \alpha y+(1-y)f\left(w_{IE}x-w_{II}y+h\right)\ .
\end{aligned}
\tag{4}
$$

Note that the A's, which are the difference in transition rates, give the expected increment, or drift, in each population. The D's, which are the sums of the transition rates, will give the noise amplitude.

We use the Taylor expansions

$$
\begin{aligned}
&A_E(E+N^{-1/2}\xi_E, I+N^{-1/2}\xi_I) \\
&\qquad= A_E(E,I)+N^{-1/2}\xi_E A_{E,E}(E,I) \\
&\qquad+N^{-1/2}\xi_I A_{E,I}(E,I)+N^{-1}\frac{1}{2}\xi_E^2 A_{E,EE}(E,I) \\
&\qquad+N^{-1}\xi_E\xi_I A_{E,EI}(E,I)+\frac{1}{2}N^{-1}\xi_I^2 A_{E,II}(E,I)+\dots
\end{aligned}
\tag{5}
$$

and so on. Our aim is to expand the Master equation as a Taylor series in (ξ_E, ξ_I) about the deterministic solution (E, I). First note that, because of the factor $N^{1/2}$ in $k = NE + N^{1/2}\xi_E$, $l = NI + N^{1/2}\xi_I$, we may write

$$
\begin{aligned}
\frac{\partial}{\partial \xi_E} &= N^{1/2}\frac{\partial}{\partial k} \\
\frac{\partial}{\partial \xi_I} &= N^{1/2}\frac{\partial}{\partial l}
\end{aligned}
\tag{6}
$$

We replace the probability distribution over the original variables, $p_{k,l}(t)$, with a distribution over the fluctuations, $\Pi(\xi_E, \xi_I, t)$. The time derivatives are related via

$$\begin{aligned} \partial_t p_{k,l}(t) &= \partial_t \Pi(\xi_E, \xi_I, t) + \partial_t \xi_E \partial_{\xi_E} \Pi + \partial_t \xi_I \partial_{\xi_I} \Pi \\ &= \partial_t \Pi(\xi_E, \xi_I, t) - N^{1/2} \partial_t E \partial_{\xi_E} \Pi - N^{1/2} \partial_t I \partial_{\xi_I} \Pi \end{aligned} \tag{7}$$

where the second substitution comes from the fact that (E, I) and $N^{-1/2}(\xi_E, \xi_I)$ are both time-dependent variables, yet their sum (k, l) is independent of time in the sense that $(\partial_t k, \partial_t l) = (0, 0)$. Simultaneously expanding p, and the A's and D's, the terms of order $N^{1/2}$ give rise to equations for the deterministic terms

$$\begin{aligned} \frac{dE}{dt} &= A_E(E, I) = -\alpha E + (1 - E) f(s_E) \\ \frac{dI}{dt} &= A_I(E, I) = -\alpha I + (1 - I) f(s_I) \end{aligned} \tag{8}$$

which are the Wilson-Cowan equations [2].

The fluctuation distribution $\Pi(\xi_E, \xi_I; t)$ obeys the partial differential equation

$$\begin{aligned} \partial_t \Pi = &- A_{E,E} \partial_{\xi_E} [\xi_E \Pi] - A_{E,I} \partial_{\xi_E} [\xi_I \Pi] - A_{I,E} \partial_{\xi_I} [\xi_E \Pi] \\ &- A_{I,I} \partial_{\xi_I} [\xi_I \Pi] + \frac{1}{2} D_E {\partial_{\xi_E}}^2 \Pi + \frac{1}{2} D_I {\partial_{\xi_I}}^2 \Pi \\ &- N^{-1/2} \frac{1}{2} \left\{ A_{E,EE} \partial_{\xi_E} \left[\xi_E^2 \Pi\right] + 2 A_{E,EI} \partial_{\xi_E} [\xi_E \xi_I \Pi] \right. \\ &\quad \left. + A_{E,II} \partial_{\xi_E} \left[\xi_I^2 \Pi\right] \right\} \\ &- N^{-1/2} \frac{1}{2} \left\{ A_{I,EE} \partial_{\xi_I} \left[\xi_E^2 \Pi\right] + 2 A_{I,EI} \partial_{\xi_I} [\xi_E \xi_I \Pi] \right. \\ &\quad \left. + A_{I,II} \partial_{\xi_I} \left[\xi_I^2 \Pi\right] \right\} \\ &+ N^{-1/2} \frac{1}{2} \left\{ D_{E,E} {\partial_E}^2 [\xi_E \Pi] + D_{E,I} {\partial_E}^2 [\xi_I \Pi] \right\} \\ &+ N^{-1/2} \frac{1}{2} \left\{ D_{I,E} {\partial_E}^2 [\xi_E \Pi] + D_{I,I} {\partial_E}^2 [\xi_I \Pi] \right\} \\ &- N^{-1/2} \frac{1}{6} \left\{ A_E {\partial_E}^3 \Pi + A_I {\partial_I}^3 \Pi \right\} + O(N^{-1}) \end{aligned} \tag{9}$$

where $A_{E,IE} = \partial_{\xi I} \partial_{\xi_E} A_E$, and so on, are all evaluated at the solutions (E, I) of (8). These equations generalize the one population system-size expansion of the master equation reported in [3]. See also [4].

If we drop terms of $O(N^{-1/2})$ and smaller, then equation (9) becomes a Fokker-Planck equation with linear drift term and additive noise. This is called the linear noise approximation and its solutions are Gaussian [1]. It is more transparent to write the linear noise approximation as the equivalent Itô form Langevin equation

$$\frac{d}{dt} \begin{pmatrix} \xi_E \\ \xi_I \end{pmatrix} = A \begin{pmatrix} \xi_E \\ \xi_I \end{pmatrix} + \begin{pmatrix} \sqrt{D_E} \eta_E \\ \sqrt{D_I} \eta_I \end{pmatrix} \tag{10}$$

where the A terms are from the Jacobian matrix of the Wilson-Cowan equations

$$A(E, I) = \begin{pmatrix} A_{E,E}(E, I) & A_{E,I}(E, I) \\ A_{I,E}(E, I) & A_{I,I}(E, I) \end{pmatrix} \tag{11}$$

where

$$A_{E,E}(E,I) = -\alpha - f(s_E) + (1-E)w_{EE}f'(s_E) \tag{12}$$
$$A_{E,I}(E,I) = -(1-E)w_{EI}f'(s_E) \tag{13}$$
$$A_{I,E}(E,I) = (1-I)w_{IE}f'(s_I) \tag{14}$$
$$A_{I,I}(E,I) = -\alpha - f(s_I) - (1-I)w_{II}f'(s_I)\ . \tag{15}$$

The η_E and η_I are independent white noise variables with variance 1, and the D terms governing the noise amplitude in (10) are given by

$$\begin{aligned} D_E(E,I) &= \alpha E + (1-E) f\left(w_{EE}E - w_{EI}I + h_E\right) \\ D_I(E,I) &= \alpha I + (1-I) f\left(w_{IE}E - w_{II}I + h_I\right)\ . \end{aligned} \tag{16}$$

All the terms in (10) are evaluated at the deterministic solution, (E,I).

If the population sizes are not equal, the expansion carries through in essentially the same way, using two small parameters $N_E^{-1/2}$ and $N_I^{-1/2}$. We start with the decomposition $k = N_E\tilde{E} = N_E E + N_E^{1/2}\xi_E$, $l = N_I I + N_I^{1/2}\xi_I$, and similarly derive equations for the evolution of the deterministic part (E,I) and the fluctuating part (ξ_E,ξ_I). A key difference is

$$\begin{aligned} \frac{\partial}{\partial \xi_E} &= N_E^{1/2}\frac{\partial}{\partial k} \\ \frac{\partial}{\partial \xi_I} &= N_I^{1/2}\frac{\partial}{\partial l}\ . \end{aligned} \tag{17}$$

If the populations are very different in size, for example $N_E = 4N_I$ in a cortical column [5], then we expect the perturbation series in the larger excitatory population to converge more quickly, and so the inhibitory population would have a larger direct noise influence.

S1.2 Avalanche case

Given now the symmetric conditions $w_{IE} = w_{EE} = w_E$, $w_{EI} = w_{II} = w_I$, and $h_E = h_I = h$, we make a change of variables to the mean Σ and difference Δ where

$$\begin{pmatrix}\Sigma \\ \Delta\end{pmatrix} = \begin{pmatrix}1/2 & 1/2 \\ 1/2 & -1/2\end{pmatrix}\begin{pmatrix}E \\ I\end{pmatrix} \tag{18}$$

as in [6]. In this rotated frame the deterministic equations become

$$\begin{aligned} \frac{d\Sigma}{dt} &= -\alpha\Sigma + (1-\Sigma)f(s) \\ \frac{d\Delta}{dt} &= -\Delta\left(\alpha + f(s)\right) \end{aligned} \tag{19}$$

which has unique stable solution $(\Sigma_0, 0)$. The factor of Δ in the equation for $\frac{d\Delta}{dt}$ ensures that the difference is zero at the fixed point, in other words the activity in both populations is equal, i.e. $E_0 = I_0 = \Sigma_0$. Note that the input

$$\begin{aligned} s &= w_E E - w_I I + h \\ &= (w_E - w_I)\Sigma + (w_E + w_I)\Delta + h \end{aligned} \tag{20}$$

is the same for both populations, in this symmetric case.

Similarly, equation (10) becomes

$$\frac{d}{dt}\begin{pmatrix}\xi_\Sigma\\ \xi_\Delta\end{pmatrix} = \tilde{A}\begin{pmatrix}\xi_\Sigma\\ \xi_\Delta\end{pmatrix} + \begin{pmatrix}1/2 & 1/2\\ 1/2 & -1/2\end{pmatrix}\begin{pmatrix}\sqrt{D_E}\eta_E\\ \sqrt{D_I}\eta_I\end{pmatrix} \tag{21}$$

where

$$\tilde{A} = \begin{pmatrix}1/2 & 1/2\\ 1/2 & -1/2\end{pmatrix} A \begin{pmatrix}1 & 1\\ 1 & -1\end{pmatrix} \tag{22}$$

After an initial transient, the solution settles near the fixed point $(\Sigma, \Delta) = (\Sigma_0, 0)$. In that case, the Jacobian (22) simplifies to the upper triangular form

$$\tilde{A} = \begin{pmatrix}-\lambda_1 & w_{ff}\\ 0 & -\lambda_2\end{pmatrix} . \tag{23}$$

Meanwhile, equation (19) tells us that $(1 - \Sigma_0)f(s_0) = \alpha\Sigma_0$, so that

$$D_E = D_I = \alpha\Sigma_0 + (1 - \Sigma_0)f(s_0) = 2\alpha\Sigma_0 \tag{24}$$

and, because the sum and difference of independent Gaussians with equal variance are both independent Gaussians, the noise term is

$$\begin{pmatrix}1/2 & 1/2\\ 1/2 & -1/2\end{pmatrix}\begin{pmatrix}\sqrt{D_E}\eta_E\\ \sqrt{D_I}\eta_I\end{pmatrix} = \sqrt{\alpha\Sigma_0}\begin{pmatrix}\eta_\Sigma\\ \eta_\Delta\end{pmatrix} \tag{25}$$

where η_Σ and η_Δ are independent white noise variables. So, the linear noise approximation in the mean and difference variables is considerably simplified as

$$\frac{d}{dt}\begin{pmatrix}\xi_\Sigma\\ \xi_\Delta\end{pmatrix} = \begin{pmatrix}-\lambda_1 & w_{ff}\\ 0 & -\lambda_2\end{pmatrix}\begin{pmatrix}\xi_\Sigma\\ \xi_\Delta\end{pmatrix} + \sqrt{\alpha\Sigma_0}\begin{pmatrix}\eta_\Sigma\\ \eta_\Delta\end{pmatrix} . \tag{26}$$

The upper triangular matrix, $\tilde{A}$, is the functionally feedforward structure: the value of ξ_Δ feeds forward into the evolution of ξ_Σ but the value of of ξ_Σ does not directly feed back into the evolution of ξ_Δ.

S1.2.1 Moment Equations for Avalanche case

From (9), we may write down moment equations for the fluctuations (ξ_E, ξ_I) [1]. However, it is easier to calculate the moments in the variables (Σ, Δ). The Jacobian and diffusion terms are given in (26) above. The second derivatives are

$$\begin{aligned}
\tilde{A}_{\Sigma,\Sigma\Sigma}(\Sigma,\Delta) &= -2w_0 f'(s_\Sigma) + (1-\Sigma)w_0^2 f''(s_\Sigma)\\
\tilde{A}_{\Sigma,\Sigma\Delta}(\Sigma,\Delta) &= -(w_E + w_I)f'(s_\Sigma) + (1-\Sigma)w_0(w_E + w_I)f''(s_\Sigma)\\
\tilde{A}_{\Sigma,\Delta\Delta}(\Sigma,\Delta) &= (1-\Sigma)(w_E + w_I)^2 f''(s_\Sigma)\\
\tilde{A}_{\Delta,\Sigma\Sigma}(\Sigma,\Delta) &= -w_0^2\Delta f''(s_\Delta)\\
\tilde{A}_{\Delta,\Sigma\Delta}(\Sigma,\Delta) &= -w_0 f'(s_\Delta) - w_0(w_E + w_I)\Delta f''(s_\Delta)\\
\tilde{A}_{\Delta,\Delta\Delta}(\Sigma,\Delta) &= -2(w_E + w_I)f'(s_\Delta) - (w_E + w_I)^2\Delta f''(s_\Delta)
\end{aligned} \tag{27}$$

At the fixed point $(\Sigma_0, 0)$, the last three of equations (27) simplify to

$$\begin{aligned}
\tilde{A}_{\Delta,\Sigma\Sigma}(\Sigma_0,0) &= 0\\
\tilde{A}_{\Delta,\Sigma\Delta}(\Sigma_0,0) &= -w_0 f'(s_\Delta)\\
\tilde{A}_{\Delta,\Delta\Delta}(\Sigma_0,0) &= -2(w_E + w_I)f'(s_\Delta)
\end{aligned} \tag{28}$$

where $w_0 = w_E - w_I$.

The evolution equations for the means are

$$\frac{d}{dt}\langle\xi_\Sigma\rangle = -\lambda_1\langle\xi_\Sigma\rangle + w_{ff}\langle\xi_\Delta\rangle + O(N^{-1/2})$$
$$\frac{d}{dt}\langle\xi_\Delta\rangle = -\lambda_2\langle\xi_\Delta\rangle + O(N^{-1/2}) \tag{29}$$

The second central moments have evolution equations

$$\frac{d}{dt}\mathrm{Var}(\xi_\Sigma) = -2\lambda_1\mathrm{Var}(\xi_\Sigma) + 2w_{ff}\mathrm{Cov}(\xi_\Sigma,\xi_\Delta) + \alpha\Sigma_0$$
$$\frac{d}{dt}\mathrm{Var}(\xi_\Delta) = -2\lambda_2\mathrm{Var}(\xi_\Delta) + \alpha\Sigma_0$$
$$\frac{d}{dt}\mathrm{Cov}(\xi_\Sigma,\xi_\Delta) = -(\lambda_2+\lambda_1)\mathrm{Cov}(\xi_\Sigma,\xi_\Delta) + w_{ff}\mathrm{Var}(\xi_\Delta) \tag{30}$$

to order N^0, with stationary solutions to the same order of

$$\mathrm{Var}(\xi_\Delta) = \frac{\alpha\Sigma_0}{2\lambda_2}$$
$$\mathrm{Cov}(\xi_\Sigma,\xi_\Delta) = \frac{\alpha\Sigma_0 w_{ff}}{2\lambda_2(\lambda_2+\lambda_1)}$$
$$\mathrm{Var}(\xi_\Sigma) = \frac{\alpha\Sigma_0}{2\lambda_1}\left(1+\frac{w_{ff}^2}{\lambda_2(\lambda_2+\lambda_1)}\right)\ . \tag{31}$$

S1.3 Corrections to the mean at order N^{-1}

If we add next-order terms into the equations (29), the evolution equations for the means contain correction terms at order $N^{-1/2}$

$$\begin{aligned}\frac{d}{dt}\langle\xi_\Sigma\rangle &= -\lambda_1\langle\xi_\Sigma\rangle + w_{ff}\langle\xi_\Delta\rangle \\ &+ \frac{1}{2}N^{-1/2}\left(A_{\Sigma,\Sigma\Sigma}\left\langle\xi_\Sigma^2\right\rangle + \right. \\ &+ \left. 2A_{\Sigma,\Sigma\Delta}\langle\xi_\Sigma\xi_\Delta\rangle + A_{\Sigma,\Delta\Delta}\left\langle\xi_\Delta^2\right\rangle\right) + O(N^{-1}) \\ \frac{d}{dt}\langle\xi_\Delta\rangle &= -\lambda_2\langle\xi_\Delta\rangle \\ &+ \frac{1}{2}N^{-1/2}\left(2A_{\Delta,\Sigma\Delta}\langle\xi_\Sigma\xi_\Delta\rangle + A_{\Delta,\Delta\Delta}\left\langle\xi_\Delta^2\right\rangle\right) + O(N^{-1})\end{aligned} \tag{32}$$

We will substitute the stationary solutions for the variances (31) into these, and then derive stationary solutions for the means themselves by setting their derivatives to zero. Note that to order N^0, the 2nd moments are the 2nd central moments, and that the effect of substitution will be to produce corrections to the means at order N^{-1}.

The correction term for the mean of fluctuations in the sum, ξ_Σ is

$$
\begin{aligned}
c_\Sigma &= \frac{\alpha\Sigma_0}{4}N^{-1/2}\left[A_{\Sigma,\Sigma\Sigma}\frac{1}{\lambda_1}\left(1+\frac{w_{ff}^2}{\lambda_2(\lambda_2+\lambda_1)}\right)\right. \\
&\quad \left. + \, 2A_{\Sigma,\Sigma\Delta}\frac{w_{ff}}{\lambda_2(\lambda_2+\lambda_1)} + A_{\Sigma,\Delta\Delta}\frac{1}{\lambda_2}\right] \\
&= -\frac{\alpha\Sigma_0}{4\lambda_1}N^{-1/2}\left[2w_0 f' - (1-\Sigma_0)w_0^2 f''\right] \\
&\quad - \frac{\alpha\Sigma_0 w_{ff}^2}{2\lambda_2}N^{-1/2}\left[\frac{\left(w_0 f' - (1-\Sigma_0)w_0^2 f''/2\right)}{\lambda_1(\lambda_2+\lambda_1)}\right. \\
&\quad \left. + \frac{1}{(\lambda_2+\lambda_1)}\left(\frac{1}{1-\Sigma_0} - \frac{w_0 f''}{f'}\right) - \frac{f''}{2(1-\Sigma_0)(f')^2}\right] \qquad (33)
\end{aligned}
$$

where we used the definition $w_{ff} = (1-\Sigma_0)(w_E + w_I)f'$ to simplify the expression. Similarly, the correction term for the difference Δ is

$$
\begin{aligned}
c_\Delta &= \frac{\alpha\Sigma_0}{4}N^{-1/2}\left[2A_{\Delta,\Sigma\Delta}\frac{w_{ff}}{\lambda_2(\lambda_2+\lambda_1)} + A_{\Delta,\Delta\Delta}\frac{1}{\lambda_2}\right] \\
&= -\frac{\alpha\Sigma_0}{4}N^{-1/2}\left[2w_0 f'\frac{w_{ff}}{\lambda_2(\lambda_2+\lambda_1)} + 2(w_E+w_I)f'\frac{1}{\lambda_2}\right] \\
&= -\frac{\alpha\Sigma_0 w_{ff}}{2\lambda_2}N^{-1/2}\left[\frac{w_0 f'}{(\lambda_2+\lambda_1)} + \frac{1}{(1-\Sigma_0)}\right] \qquad (34)
\end{aligned}
$$

It may be checked that these correction terms are negative, so that increasing w_{ff} lowers the mean to order N^{-1}.

References

1. van Kampen N (2007) Stochastic processes in physics and chemistry. North Holland.

2. Wilson H, Cowan J (1972) Excitatory and inhibitory interactions in localized populations of model neurons. Biophysical Journal 12: 1–24.

3. Ohira T, Cowan J (1997) Stochastic neurodynamics and the system size expansion. In: Ellacort S, Anderson I, editors, Mathematics of neural networks: models, algorithms, and applications, Springer. pp. 290-294.

4. Bressloff P (2009) Stochastic neural field theory and the system-size expansion. SIAM Journal on Applied Mathematics 70: 1488-1521.

5. Abeles M (1991) Corticonics: Neural Circuits of the Cerebral Cortex. Cambridge University Press.

6. Murphy B, Miller K (2009) Balanced Amplification: A New Mechanism of Selective Amplification of Neural Activity Patterns. Neuron 61: 635–648.

Figure Legends

Figure S1. Avalanche size and duration distributions for different time bin sizes. Avalanche distributions from a single simulation with parameter values $h_E = h_I = 0.001$, $w_0 = w_E - w_I = 0.2$, $w_E + w_I = 5.8$, and $N = 800$. Left column, $\Delta t = 0.024 = \langle ISI \rangle$; right column, $\Delta t = 1 \approx 4 \langle ISI \rangle$. Upper graphs show the distribution of avalanche size in numbers of spikes, and lower graphs show the distribution of avalanche duration, i.e. the elapsed time between the first and last spike in an avalanche, in msec. Note that the data shows power law fit in all cases, but the slope of the distribution changes with the time bin size.
